# The Role of Multiscale Protein Dynamics in Antigen Presentation and T Lymphocyte Recognition

**DOI:** 10.3389/fimmu.2017.00797

**Published:** 2017-07-10

**Authors:** R. Charlotte Eccleston, Shunzhou Wan, Neil Dalchau, Peter V. Coveney

**Affiliations:** ^1^Centre for Computational Science, Department of Chemistry, University College London, London, United Kingdom; ^2^Microsoft Research, Cambridge, United Kingdom

**Keywords:** pathway model, binding affinity, machine learning, molecular dynamics, MHC-I antigen presentation pathway

## Abstract

T lymphocytes are stimulated when they recognize short peptides bound to class I proteins of the major histocompatibility complex (MHC) protein, as peptide–MHC complexes. Due to the diversity in T-cell receptor (TCR) molecules together with both the peptides and MHC proteins they bind to, it has been difficult to design vaccines and treatments based on these interactions. Machine learning has made some progress in trying to predict the immunogenicity of peptide sequences in the context of specific MHC class I alleles but, as such approaches cannot integrate temporal information and lack explanatory power, their scope will always be limited. Here, we advocate a mechanistic description of antigen presentation and TCR activation which is explanatory, predictive, and quantitative, drawing on modeling approaches that collectively span several length and time scales, being capable of furnishing reliable biological descriptions that are difficult for experimentalists to provide. It is a form of multiscale systems biology. We propose the use of chemical rate equations to describe the time evolution of the foreign and host proteins to explain how the original proteins end up being presented on the cell surface as peptide fragments, while we invoke molecular dynamics to describe the key binding processes on the molecular level, including those of peptide–MHC complexes with TCRs which lie at the heart of the immune response. On each level, complementary methods based on machine learning are available, and we discuss the relationship between these divergent approaches. The pursuit of predictive mechanistic modeling approaches requires experimentalists to adapt their work so as to acquire, store, and expose data that can be used to verify and validate such models.

## Introduction

The immune system’s ability to fight against pathogens such as viruses and bacteria varies between individuals and is influenced by an area of the human genome known as the major histocompatibility complex (MHC). MHC class I (MHCI) complexes present small fragments of proteins, known as peptides, on the cell surface, which allows cytotoxic T-cells to recognize intracellular pathogens.

**Figure 1:**
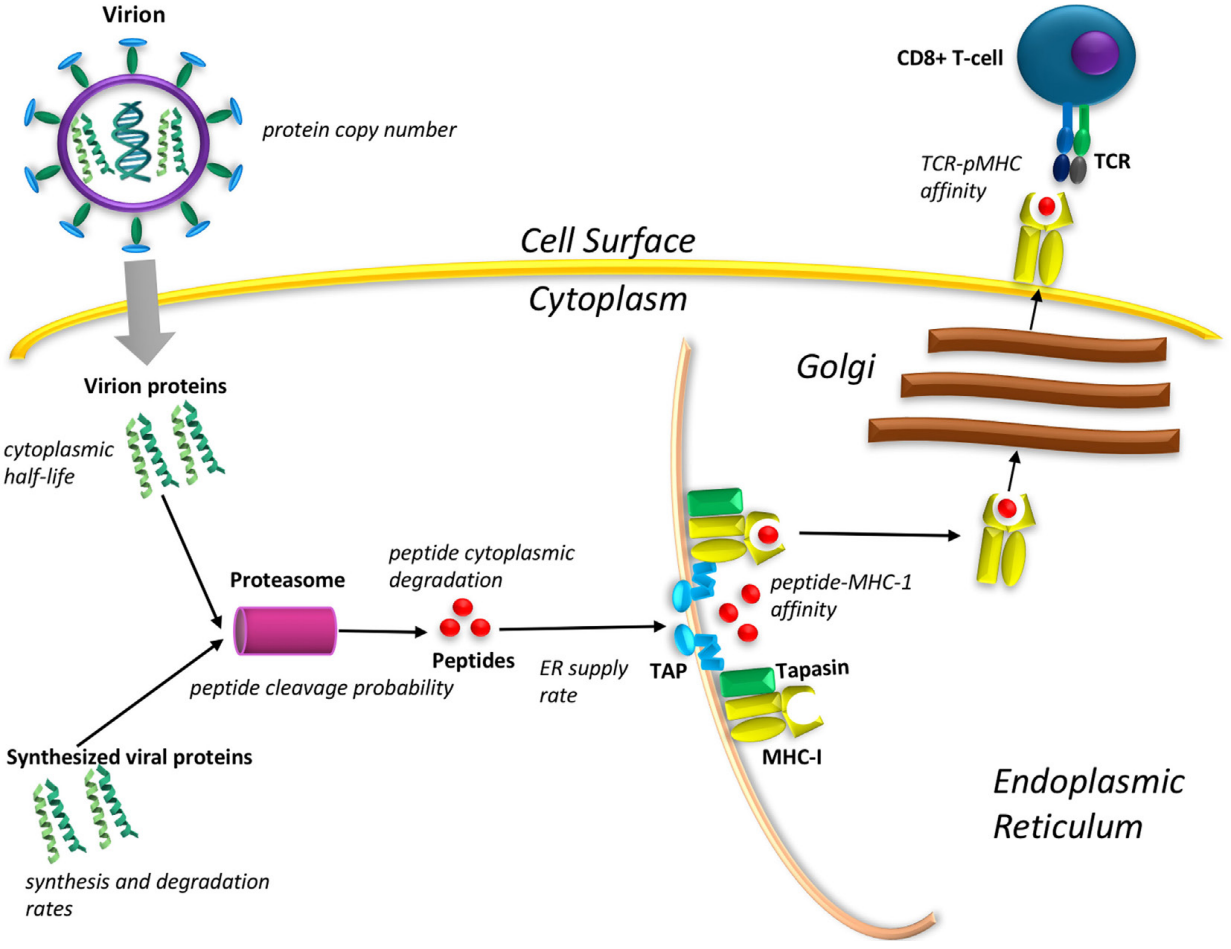
Diagrammatic representation of the major steps in the major histocompatibility complex (MHC) antigen presentation pathway that need to be included in a mechanistic model of viral peptide cell surface presentation. The measurable quantities required for such a model are mentioned in italics next to each step.

The MHCI antigen presentation pathway is a multistage process, which essentially hijacks the waste disposal system of cells (Figure 1). The proteasomal degradation of cytoplasmic proteins generates peptides that bind to the transporter associated with antigen processing (TAP), thereby shipping them to the endoplasmic reticulum (ER). Once in the ER, peptides may be loaded on to MHCI molecules, which are then transported through the Golgi apparatus to the cell surface. The abundance of specific peptides on the cell surface therefore depends on several factors. First, 99% of all cytoplasmic peptides are degraded before encountering TAP (1) so a high cytoplasmic peptide concentration is key to eventual peptide presentation. The outcome depends upon a trade-off between protein synthesis and degradation including the probability of cleavage of a peptide from the protein. The rate of transport of a peptide to the ER depends upon the sequence-specific affinity of the peptide with TAP. Once in the ER, peptides will compete for loading onto the MHC molecules *via* the peptide loading complex. Chaperone molecules, such as tapasin, facilitate the formation of peptide–MHC (pMHC) complexes with high affinity, which then egress to the cell surface. The cell surface pMHC complexes bind with T-cell receptors (TCRs), initiating a signal cascade resulting in T-cell activation and the killing of target cells. pMHC affinity to TCR (2) and cell surface peptide abundance are correlated with T-cell immunodominance (3), the dominant clonal expansion of T-cells that respond to specific peptides, or *epitopes*.

Knowledge of the hierarchy and timing of presentation of such epitopes by MHC is key to the development of peptide vaccines and immunotherapy for a myriad of diseases including viral infections and cancer. This requires measurements of T-cell recognition and/or the pMHC cell surface abundance. T-cell epitopes can be mapped using high-throughput experimental methods. However, the number of proteins and MHC alleles that can be scanned at any given time is limited. Croft et al. (4) made temporal measurements of the cell surface abundance of eight vaccinia peptides using mass spectrometry, demonstrating how the abundance of viral peptides relates to the timing of T-cell responses. However, it is infeasible at present to perform such an experiment over the full potential peptidome of viral peptides for any MHC allele. To obtain a more comprehensive view of the dynamics of viral peptide presentation, quantitative models are required to generate predictions of cell surface presentation of viral or cancerous peptides.

It is desirable to predict which peptides are *immunogenic*, that is those which evoke an immune response. Several epitope prediction algorithms have been produced using machine learning methods, such as the MHC peptide processing tool from the immune epitope database (IEDB) (5). This algorithm is built upon datasets of *in vitro* experiments characterizing proteasomal cleavage rates, TAP affinity, and MHC binding of thousands of different peptides, combining the three metrics to produce a total “score” for each possible peptide from an input protein amino acid sequence. The higher the score, the greater the probability of the peptide being presented.

Machine learning algorithms are thought to be able to predict the efficiency of peptide processing for MHC presentation accurately when comparing peptides originating from a single protein. However, their predictions provide a static view of immunogenicity based upon sequence-specificity; they cannot account for protein abundance kinetics, which has a substantial impact on the hierarchy of peptide abundance at the cell surface (4). This is a general limitation of data-driven, as opposed to theory-led, approaches in biomedical research (6). Predicting the timing and hierarchy of peptide presentation following pathogen infection requires mechanistic models that integrate pathogen kinetics throughout infection and replication. It is, however, possible to include machine learning methods within mechanistic pathway prediction models by incorporating sequence-specific distinctions between peptides *via* their kinetic behavior.

## A Motivating Example: HIV Infection and Long-Term Control

HIV-infected individuals usually progress to AIDS within 10 years, with 10−15% of people progressing rapidly within 3 years of infection, whereas 5−10% remain asymptomatic for over 10 years (7). These widely differing rates of progression are linked to the differing expression of specific MHC alleles, which in humans are known as human leukocyte antigen (HLA) proteins, and the peptides they present. Experimental evidence suggests an association between T-cell recognition of Gag epitopes presented by a subsection of MHC alleles known as long-term non-progressors (LTNPs) and control of HIV progression; however, this is far from a solved issue.

The MHC alleles HLA-B*58, -B*57, -B*27, and -B*44 are overrepresented among LTNPs and are associated with Gag-specific T-cell responses (8). Conversely, the alleles HLA-B*35 and -B*18 have been found to be associated with rapid progression to AIDS with T-cell responses against non-Gag epitopes, such as those from the Nef and Env proteins (9).

The Env and Nef proteins are both highly variable, with Env being the most variable sequence in the HIV genome (8) and mutations in these epitopes are fitness neutral (10). The Gag protein amino acid sequence, however, is highly conserved, and escape mutations in its epitopes negatively impact viral fitness. For example, the T242N escape mutation in the HLA-B*57/B*58:01 restricted Gag epitope TW10 (TSTLQEQIGW) leads to diminished viral replication capacity, as does the A163G mutation of the similarly restricted Gag KF11 (KAFSPEVIPMF) epitope (11). However, other highly conserved proteins, such as Pol, are not as strongly associated with HIV control. Therefore, sequence conservation may not be the only important factor in immune control. Gag is the most abundant protein in both the HIV virion and in the cytoplasm following the nuclear export of full-length mRNA during replication. The kinetics of the Gag protein could also be very important in shaping the resulting abundance of derived peptides. As existing analyses considering several different factors separately have yet to elucidate a coherent picture of HIV control, we propose analyzing this question in an integrated manner. Specifically, a dynamic, mechanistic model could help to determine the relative importance of protein abundance and sequence variability, and help to explain Gag’s role in the control of HIV.

## Mechanistic Pathway Models, Their Construction, and the Data Required

To create a mechanistic model to predict peptide cell surface presentation following viral infection, each step in the pathway from viral protein synthesis to pMHC binding and presentation (Figure 1) can be represented in the form of an ordinary differential equation based on the law of mass action. The rate coefficients in such equations require collection of experimental data pertaining to the viral intracellular dynamics. When a virion enters a cell, viral proteins and the viral genome are dumped into the cytoplasm. The viral proteins are degraded into peptides that can potentially lead to host recognition of the infected cell soon after infection. Therefore, determining the copy number of the viral proteins contained within a virion and their cytoplasmic half-lives are required to predict the production of virion-derived peptides prior to the onset of viral replication. Detecting infected cells before viral replication begins would prevent the spread of the virus to other cells in the body.

As mentioned above, an ideal candidate pathogen to test this method is HIV-1, as there is a wealth of experimental data available characterizing many of the important steps in viral replication within a single infected cell. Furthermore, several models of HIV-1 intracellular kinetics exist describing the dynamics of viral mRNA production together with synthesis and degradation of many of the important HIV-1 proteins, as well as steps in assembly of virion particles.

Reddy and Yin (12) modeled HIV-1 intracellular kinetics from reverse transcription and integration of the viral genome into the host genome, transcription of full-length viral mRNA, subsequent splicing, and export into the cytoplasm, followed by the translation of the proteins Gag, GagPol, and Env. Several steps in the creation of new virions are also modeled, including posttranslation protein modifications, transport to the cell membrane, and the budding and assembly of viral particles. The majority of the kinetic rate parameters used in this model have been experimentally measured, and its predictions agree well with available experimental measurements of HIV DNA, mRNA, protein, and virion concentrations.

Kim and Yin (13) also described the translation and splicing of HIV mRNA, and nuclear export; furthermore, their model includes the translation of the regulatory proteins Tat and Rev as well as their regulation of transcription and export of viral mRNA. Wang and Lai (14) modeled the transcription of viral mRNA, translation of Gag, Tat, and the accessory protein Vif, the creation of Vif-Gag, Vif-APO, and Gag-APO complexes, where APO is a cellular antiviral protein, together with incorporation of these complexes into budding virions. The model was verified using several experimental data sets. The combination of these models may be expected to result in a quantitative description of intracellular HIV-1 kinetics from viral genome integration to budding of new virions. Missing parameters could be taken from publications where available, or otherwise chosen to have plausible values.

The steps of the peptide processing pathway would also need to be converted to a kinetic pathway model. Dalchau et al. (15) presented such a model of peptide–MHC binding in the ER and subsequent egression to the cell surface while also accounting for the effect of the chaperone molecule tapasin, which enhances the preferential selection of peptides that form stable complexes with MHC. In this model, a peptide *P_i_* is supplied to the ER with rate coefficient *g_i_* where it can bind to empty MHC with rate coefficient *b* or to tapasin-bound MHC with a higher value *c*. The values for *b* and *c* are assumed to be similar for all peptides binding to the same MHC allele. The peptide can unbind from the MHC or MHC–tapasin complexes with rate coefficient *u_i_* or *u_i_*⋅*q* respectively, where *q* is the increase in the peptide–MHC unbinding rate coefficient *u_i_* in the presence of tapasin.

To combine a model of viral intracellular kinetics with a peptide processing model and simulate such a model for a large set of possible peptides requires sequence-specific parameters describing the rate of proteasomal cleavage, TAP transport and peptide–MHC binding and unbinding. The IEDB MHC processing tool (5) may be used to infer relative parameters for each peptide sequence in the model. The proteasomal cleavage prediction is a relative score proportional to the logarithm of the amount of peptide generated from the cleavage of the peptides C-terminal. The TAP transport prediction is given in terms of the IC_50_ of the interaction between TAP and a peptide. Similarly, the MHC binding prediction is given as an IC_50_ value. The proteasomal cleavage score from the IEDB machine learning tool can be converted to a relative probability of peptide production each time a protein degrades by scaling the scores so that they lie within a reasonable range of probabilities.

The predicted peptide–MHC IC_50_ can be used to approximate the dissociation constant *K_D_*=*u_i_*/*b* of the peptide when bound to MHC. As peptide off-rates are known to vary more than peptide on-rates (16), we suggest making the assumption that the variation in predicted IC_50_ values is accounted for purely in terms of variation in the peptide off-rates. Accordingly, the on-rate would be non-peptide-specific and can be assigned a value in the middle of what has been measured experimentally. In this way, peptide off-rates can be calculated from predicted IC_50_ values as *k*_off_ = *k*_on_ × IC_50_. For example, by using *k*_on_ = 10^4^ M^−1^ s^−1^ [in the middle of values measured for peptides at 26°C and 32°C in the study by Garstka et al. (16)], a tight binding peptide with affinity 1 nM would translate into an off-rate of *k*_off_ = 10^−5^ s^−1^.

However, not all presented peptides will become T-cell epitopes. Calis et al. (17) used a large dataset of immunogenic and non-immunogenic pMHC complexes to determine the important amino acid properties associated with immunogenic pMHCs, and trained a predictive model to classify new pMHCs as either immunogenic or non-immunogenic. Therefore, a final step in the large mechanistic predictive model would be to use predictions of pMHC immunogenicity to filter for those peptides that are predicted to be presented that will actually result in a T-cell response. A further step involves combining existing models of T-cell signaling in response to pMHC complexes—the most recent being Lever et al. (2)—to further investigate the differences in predicted T-cell response to different pMHC complexes.

## Molecular Dynamics Studies of TCR–pMHC Interactions

A signal cascade involves a series of communications among a number of proteins and small molecules which establish many interactions within and between signaling networks. The interactions induce conformational changes that are important to many aspects of protein function. TCR interacting with antigenic peptides bound to MHC molecules, for example, changes the conformations of both pMHC and TCR, and initiates a signal cascade. There is a huge gap between the atomic-resolution molecule–molecule interactions and the cellular or intercellular level of signaling, which is both spatial and temporal (18). A large number of parameters are required in the pathway models (these are primarily rate coefficients) and for the most part they are difficult, if not impossible, to measure, or infer from data. Molecular dynamics (MD) simulations (19), along with multiscale modeling, provide a way to estimate the kinetic parameters for the pathway modeling (18, 20).

T-cell receptor–pMHC recognition involves two steps: a peptide binds to an MHC molecule to create a peptide–MHC complex, the complex being presented to TCRs. X-ray crystallographic structures provide detailed insights into the TCR–pMHC interactions, the number of structures having increased significantly in recent years. However, there are two major limitations: (i) they provide static pictures of “snapshots” from a vast ensemble of dynamic conformations; (ii) they reveal a partial representation of a full complex, usually its extracellular domain, due to the problem of crystallizing the membrane-associated proteins. Experimental methods have been used to characterize TCR–pMHC interactions, including surface plasmon resonance, IC_50_, micropipette adhesion frequency, and atomic force microscopy.

A range of immunoinformatics (i.e., machine learning)-based binding affinity prediction methods have also been developed, of which sequence-based and structure-based methods are two main classes with variable accuracies, usually depending on the size and quality of training sets (5, 6, 21, 22). As in the context of pathway modeling, a mechanistic, and quantitative approach exists for obtaining predictions of pMHC or TCR–pMHC binding affinities, based on MD simulation (23–25). It has the advantage that it provides *de novo* prediction of all relevant properties based on certain prior information.

The MD approach (19), and molecular modeling in general, is valuable to elucidate the molecular structures, dynamics, and function of biological molecules. Indeed, the Nobel Prize for Chemistry was awarded to Karplus, Levitt, and Warshel in 2013 for their work on multiscale modeling of biological systems (26). MD is a computer simulation technique wherein the time evolution of a set of interacting atoms is followed by integrating their Newtonian equations of motion. MD simulations can, in principle, provide “an unsurpassed and unsurpassable level of detail” (23) of dynamic phenomena, one that can greatly enhance our understanding of biological function. Today, MD is feasible for very complex macromolecules, such as multi-protein complexes in a heterogeneous environment which can consist of water molecules, ions, lipid bilayers, etc.

**Figure 2:**
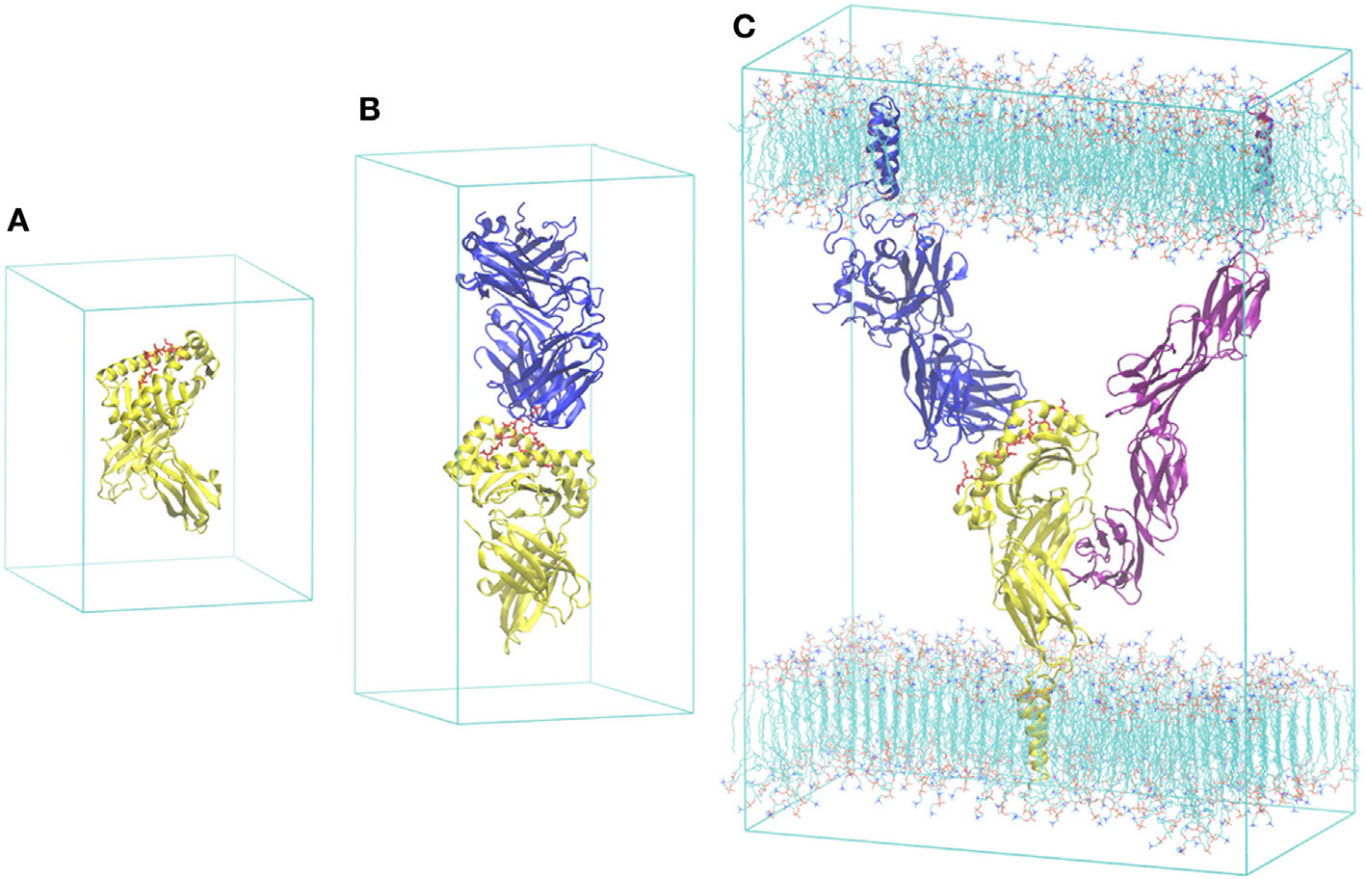
The escalating scale necessary to simulate large-scale immunological phenomenon: **(A)** model of the peptide-–MHC (pMHC) (28, 29), **(B)** pMHC bound to T-cell receptor (TCR) (30, 31), and **(C)** a viable unit of the immune synapse, comprising pMHC, TCR, CD4, and two opposing sections of membrane (32). Major histocompatibility complex (MHC) is colored yellow, TCR blue, the peptide red, and the CD4 purple. All of the models are simulated with explicit water molecules and 3D periodic boundary conditions. Water molecules are omitted for clarity.

Since the first simulation of a protein was published in 1977 (27), the MD methodology and its applications have progressed substantially. Applications in immunology have been summarized in a few review papers (23–25). Here, we mention some of our studies in the area, in the order of publication and the complexity of the systems investigated: (i) truncated pMHC (α1–α2 domains of MHC) (28); (ii) pMHC with entire extracellular domain of MHC (α1, α2, α3, and β2m domains of MHC) (28, 29) (Figure 2A); (iii) TCR–pMHC (entire extracellular domains of TCR and MHC) (30, 31) (Figure 2B); and (iv) TCR–pMHC–CD4 (cluster of differentiation 4) embedded in a lipid bilayer (32) (Figure 2C). These studies demonstrate that structural and energetic properties can be accurately predicted using molecular systems in realistic settings through the introduction of physiological details. In the TCR–pMHC–CD4 study (32), the tri-molecular system was constructed based on available X-ray structures with missing regions modeled by homology, including transmembrane domains, adjoining regions, and loops. The entire model was embedded in a membrane environment likely to influence the interactions of these membrane-associated proteins with each other. The computed structural and thermodynamic properties from the simulation are in good agreement with limited experimental data, including the binding free energies of CD4 to pMHC and pMHC to TCR. As we have demonstrated recently in small molecule–protein systems (33, 34), the simulations could provide insight into the interactions of individual molecule–molecule complexes. The overall free energy change upon binding, along with the kinetic properties such as the on- and off-rates, is critical for the recognition and discrimination process. The binding of pMHC to TCR is, in terms of underlying physico-chemical principles, identical to binding of small molecule inhibitors to protein receptors, although the models and simulations need to be carefully designed because of the complexity of the protein–protein complexes.

It should be noted that while the current paper focuses on MHC I-based recognition, the model of the TCR–pMHC–CD4 (32) is a CD4 T cell that interacts with peptides on MHC class II. There are no crystallographic structures reported for a TCR–pMHC–CD8 ternary complex. A hypothetical model of TCR–pMHC–CD8 was assembled based on the available structures of the components, which demonstrated remarkable similarities in the overall topology with the TCR–pMHC–CD4 complex (35). The TCR–pMHC–CD4 or TCR–pMHC–CD8 tri-molecular complex, which is a key unit for the immune synapse, provides the minimum complexity needed to trigger transient calcium signaling. The simulation of the TCR–pMHC–CD4 model (32) therefore provided a basis for understanding how the CD4 and CD8 act as co-receptors during the process of T lymphocyte recognition. The immune synapse involves the formation of a highly organized pattern of proteins in the intercellular junction, of which the pattern is spontaneous evolving. This is another aspect in the multiscale process, resulting from self-assembly processes and active feedback mechanisms. Coarse-grained descriptions, such as a mathematical representation using reaction–diffusion equations (36), can be used to model the synapse formation.

Molecular dynamics methods have been developed for the improvement of configurational sampling, of which replica-exchange (37) and biased potential (38) methods are among the more promising. Our recent work shows that ensemble-based methods are capable of producing rapid, accurate, precise, and reliable binding free energies (33, 34, 39, 40). In these studies, we have used these approaches, termed “enhanced sampling of molecular dynamics with approximation of continuum solvent” (ESMACS) (40) and “thermodynamic integration with enhanced sampling” (TIES) (39). Even for peptide–MHC molecular systems where peptides are much larger, more flexible, and diverse than most small molecules used in drug discovery, ESMACS produces precise and reproducible free energy estimates, which correlate well with experimental data (40). Using standard protocols as established in our publications (33, 34, 39, 40), reproducible results can be generated with MD for molecular systems as complicated as the multi-protein complexes implicated in the immune response.

Recent models of MHCI presentation have predicted a beneficial role for conformational flexibility in shaping the dynamics of peptide loading (29). This work demonstrated that pathway models explicitly describing an encounter complex during peptide–MHC loading were most predictive of experimental observations. In the same study, MD analysis showed evidence of multiple conformations in some but not all MHC alleles. The hierarchy of conformational flexibility observed by MD was the same as the ordering of rate constants in the pathway model, demonstrating consistency in the two modeling approaches, despite the analyses being aimed at timescales differing by many orders of magnitude. Moreover, there is scope to use MD to generate estimates for key missing parameters, most notably peptide–MHCI off-rates, as experimental measurements are only available for a very small subset of the peptidome, to complement or extend immunoinformatics methods.

## Agent-Based Models Can Integrate Intracellular and Intercellular Interactions

For a long time now, there have been attempts to model and simulate the interactions of the multiple cell types that contribute to immune responses (41). However, only more recently have truly multiscale models emerged, where both the intracellular biochemistry and cell–cell interactions can be analyzed simultaneously (42). This is due, in part, to advances in computer hardware. However, simulation of such models remains cumbersome, as each cell requires a set of sizeable differential equations to be numerically integrated, leading to a very large number of such equations overall. Accordingly, parameter inference of agent-based models is normally impractical. Instead, the intracellular biochemistry can be modeled in isolation first, and calibrated against data from isogenic cultures. Furthermore, such isolation would enable the intracellular models to be simplified, making their embedding in agent-based simulations less computationally demanding.

Applied to HIV, agent-based modeling could be instrumental in understanding the contributions of direct- and cross-presentation to T-cells, which has been the subject of much debate (43). Many of the components required to build such a model already exist, but would need to be adapted and calibrated to experimental data. In Section “A Motivating Example: HIV Infection and Long-term Control,” we described how direct-presentation in HIV-infected cells can be modeled at the cellular level. Adapting the model of MHCI presentation to dendritic cells could be achieved by redefining the peptide supply terms (*g_i_*) to reflect internalization and processing of extracellular antigen. How the new values of *g_i_* are to be specified would require further work, but relevant data are already available [e.g., Ref. (44)]. Finally, interactions between dendritic cells and T-cells have already been modeled in multicellular simulations (45), offering a starting point for creating an integrated model of direct- and cross-presentation.

## Concluding Remarks

As we have discussed elsewhere, biology and medicine are dominated by the primacy of experimental observations, where theory is essentially a form of rationalization invoked to explain observations *post hoc* (6). Scientific progress, based in part on the increasing acquisition of all forms of data, and the pressing need to make sense of it, has now reached a stage wherein predictive, mechanistic, and quantitative modeling methods are emerging and could transform the future of these disciplines. The accessibility and accuracy of the theoretical predictions, for example, in clinically relevant HIV drug ranking (46, 47) and in pharmaceutical compound evaluation (33, 34), support our perspective about predictive computational modeling in pharmaceutical drug discovery and personalized medicine. In order that these methods realize their full potential, especially for personalized medicine, it is imperative that biologists design their experiments to facilitate the construction and exploitation of quantitative models (48). Immunology, with its intrinsically multiscale nature, is a field which stands to benefit greatly from this new approach.

## Author Contributions

All authors designed the research and contributed in the writing of the paper.

## Conflict of Interest Statement

The authors declare that the research was conducted in the absence of any commercial or financial relationships that could be construed as a potential conflict of interest. The handling editor declared a past coauthorship with one of the authors, ND, and states that the process nevertheless met the standards of a fair and objective review.
